# Hydroethanolic Leaf Extract of *Cordia vignei* Hutch and Dalziel Inhibits Carrageenan-Induced Foot Oedema in Chicks, Prostaglandin E_2_-Induced Paw Oedema in Mice, and Bradykinin-Induced Paw Oedema in Mice

**DOI:** 10.1155/2023/9525015

**Published:** 2023-04-07

**Authors:** George Owusu, George K. Ainooson, Newman Osafo

**Affiliations:** ^1^Deparment of Medical Laboratory Science, University of Energy and Natural Resources, Sunyani, Ghana; ^2^Department of Pharmacology, Faculty of Pharmacy and Pharmaceutical Sciences, Kwame Nkrumah University of Science and Technology, Kumasi, Ghana

## Abstract

**Background:**

*Cordia vignei* Hutch and Dalziel (Fam. Boraginaceae) is a woody plant found in west tropical Africa. The aim of this research is to find out if the leaf extract of this plant prevents oedema in animal models.

**Methods:**

(a) Inflammation was induced in the animals by injecting 100 *µ*l of 2% lambda carrageenan into the subplantar tissue of the right footpads of 7-day-old chicks 1 h before or after oral administration of 30–300 mgkg^−1^ CVE. Oedema was measured for 5 h using the water displacement method. (b) Oedema was induced in ICR mice by subplantar injection of prostaglandin E2 (PGE_2_) (50 *µ*l of 1 nM) 30 minutes before or after CVE administration. Oedema was measured for 3 h. (c) Oedema was induced in ICR mice by subplantar injection of bradykinin (BK) (10 nmol/paw) 30 min before or after administration of extract.

**Results:**

We found that CVE significantly (*P* < 0.05) prevented inflammation that was induced by injecting carrageenan into the footpads of the chicks. Also, we observed that CVE prevented inflammation produced by injecting PGE_2_ into the subplantar tissue of mice. Finally, we also report that CVE prevented inflammation produced by injecting BK into the subplantar tissues of mice. All these effects were observed in both preventive and curative protocols.

**Conclusion:**

We conclude that *Cordia vignei* leaf extract has potential anti-inflammatory activity.

## 1. Introduction

Inflammation is a process by which the body's immune system reacts to the destruction of tissues caused by chemical, physical, or microbial factors. It is the normal physiological attempt of the body to repair injuries and defend against foreign invasions [1]. Inflammatory response involves recruitment of cells including neutrophils and tissue macrophages that release mediators to enhance vasodilation, vascular permeability, and exudation of fluid into the site of injury or infection and these are responsible for the swelling [2, 3].

Presently, NSAIDs are the main therapies for inflammatory conditions [4]. However, due to the side effects of these conventional drugs, natural products that have potential anti-inflammatory activity are being explored globally as alternatives or supplements to standard treatment [5]. One of these useful plants in traditional medicine in Ghana is *Cordia vignei*. It is used to treat diseases such as sore, colitis, and prostate cancer [6–9]. There is limited data to support this claim. Our purpose of this research is to test the anti-inflammatory activity of hydroethanolic extract of *Cordia vignei* leaf on carrageenan-induced oedema in chicks, PGE_2_-induced paw oedema, and BK-induced paw oedema in mice.

## 2. Materials

### 2.1. Plant Collection and Extraction

Collection of the fresh leaves, authentication, and extraction were performed as we described previously [7].

### 2.2. Animals

Day-old chicks (*Gallus gallus*; Shaver 579) were purchased from Akropong Farms, Kumasi, Ghana, and kept in the laboratory for 7 days. ICR mice (8–9 weeks; 20–24 g) were obtained from the Animal House of the Department of Pharmacology, KNUST, Kumasi, Ghana.

Clean water and commercial feed containing the right proportions of nutrients were given to the animals throughout the study.

All animals were carefully handled as described in the Guide for the Care and Use of Laboratory Animals [10].

### 2.3. Drugs and Chemicals

Prostaglandin E_2,_ Bradykinin, and Lambda carrageenan (Λ-Carrageenan) were purchased from Sigma–Aldrich Chemical Co, St Louis, MO, USA. Diclofenac sodium was purchased from Tobinco pharmaceuticals Ltd, Ghana.

## 3. Method

### 3.1. Phytochemical Screening of *Cordia vignei* Extract

The extract was screened qualitatively for the presence of saponins, tannins, glycosides, alkaloids, flavonoids, steroids, and terpenoids as described, respectively, by Sofowora [11]; Usman et al. [12]; Evans [13]; Houghton and Raman [14]; Ayoola et al. [15]; and Jana and Shekhawat [16].

### 3.2. Carrageenan-Induced Foot Oedema

Seventy (70) healthy chicks were weighed and put into 14 Groups (*n* = 5). Chicks in Groups I-VII were used for prophylactic study, and chicks in groups VIII-XIV were used for curative study. The initial foot volume of each chick was measured using the water displacement method [17]. All the chicks in both prophylactic and curative studies were given injection of 100 *µ*l of 2% (w/v) lambda carrageenan into the right footpad to induce oedema.

In the prophylactic study, chicks in group I (control) were given normal saline (10 mlkg^−1^ p.o.); chicks in groups II–IV received diclofenac (10–100 mgkg^−1^ p.o.); and those in groups V–VII were treated with CVE (30–300 mgkg^−1^ p.o.) one hour before carrageenan injection. In the curative study, chicks in group VIII (control) received saline (10 mlkg^−1^ p.o.); chicks in groups IX–XI received diclofenac (10–100 mgkg^−1^ p.o.); and those in groups XII–XIV were given CVE (30–300 mgkg^−1^ p.o.) 1 h after carrageenan injection.

The foot volume of the chick was measured at every 1 h for 5 h.

The increase in foot volume was calculated as shown in the following equation:

(1) $$\begin{array}{c} \left[\frac{Fvt-Fvo}{Fvo}\right]\times 100\text{,} \end{array}$$

where *Fvt* is the foot volume of the chick at various times measured after induction and *Fvo* is the foot volume before induction.

### 3.3. Prostaglandin E_2_-Induced Paw Oedema in Mice

Thirty (30) healthy ICR mice were put into six groups (*n* = 5), and the initial right hind paw thickness of each mice was measured with a digital caliper (VC1346i, MP Lab Equip, USA).

Mice in groups I and II received saline (10 mlkg^−1^ p.o.), mice in group III orally received 10 mg/kg diclofenac, and mice in groups IV, V, and VI orally received 30, 100, and 300 mgkg^−1^ CVE, respectively. In the prophylactic study, PGE_2_ (50 *µ*l of 1 nM) was injected 30 min post-CVE administration. In the curative test, mice were given oral treatments of diclofenac or CVE 30 min after the PGE_2_ challenge. Inflammation was measured at 30 min intervals for three hours.

### 3.4. Bradykinin-Induced Paw Oedema

Mice were put into 6 groups of 5 and were given a subcutaneous injection of 5 mg/kg of captopril to inhibit kininase II activity. Mice received a subplantar injection of bradykinin (10 nmol/paw) 30 min after oral administration of saline (1 mlkg^−1^) or extract (30–100 mgkg^−1^). In the therapeutic protocol, mice were given CVE (30, 100, and 300 mgkg^−1^) orally thirty minutes after a bradykinin challenge. Inflammation was measured for three hours at thirty minutes intervals using a digital caliper.

### 3.5. Statistical Analysis

The results were presented as the mean ± SEM. GraphPad Prism for Windows version 6.01 (GraphPad Software Inc., San Diego, CA, USA) was used for all statistics. Raw values were normalized as percentages. Data with two independent variables were analyzed using a two-way ANOVA followed by Bonferroni's *post hoc* test. Data with one independent variable was analyzed using Student's *t*-test.

## 4. Results

### 4.1. Phytochemical Test

Phytochemicals present in CVE are shown in Table 1.

### 4.2. Effect of CVE on Carrageenan-Induced Foot Oedema in Chicks

**Figure 1:**
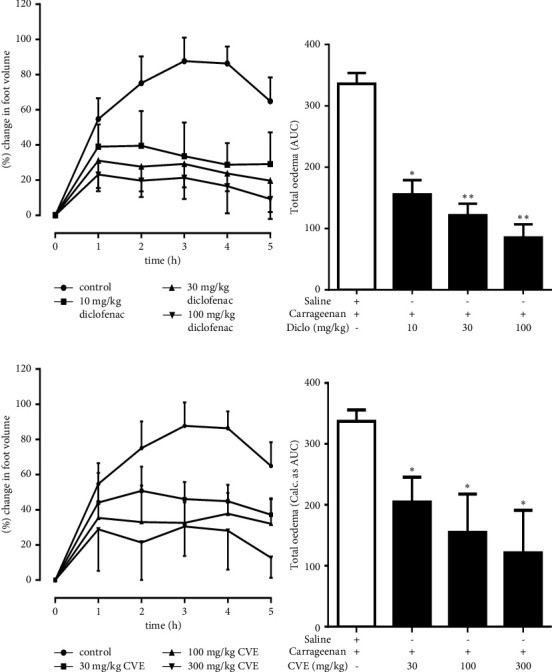
Prophylactic effect of CVE on carrageenan-induced foot oedema in chicks. Inflammation was induced as described in method. (a, b) Time-course effect and AUC of diclofenac; (c, d) Time-course and AUC of CVE. ^*∗∗*^*P* ≤ 0.05; ^*∗∗*^*P* ≤ 0.01 (control vs. treated group).

In the prophylactic study, carrageenan injection evoked a significant oedema over the 5 h period (Figures 1(a) and 1(c)). Diclofenac significantly (*P* < 0.05) reduces the total oedema from 336.30 ± 7.78 (seen in the control group) by 54.51 ± 7.14%, 64.53 ± 2.56%, and 75.03 ± 3.03%, respectively, at doses 10, 30, and 100 mgkg^−1^ (Figure 1(b)).

CVE also inhibited total oedema by 40.59 ± 4.61%, 55.29 ± 7.72%, and 64.05 ± 9.61% at doses 30, 100, and 300 mgkg^−1^, respectively, compared to the control (Figure 1(d)).

**Figure 2:**
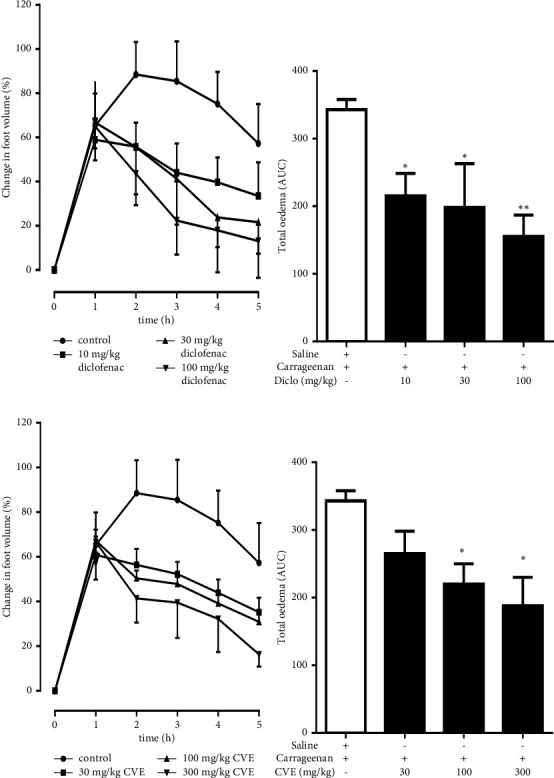
Curative effect of CVE on carrageenan-induced foot oedema in chicks. Oedema was induced in 7-day-old chicks as described in methods. (a, b) Time-course effect and AUC of diclofenac; (c, d) Time-course effect and AUC of CVE. The arrows indicate time of diclofenac or CVE administration. ^*∗*^*P* ≤ 0.05; ^*∗∗*^*P* ≤ 0.01 (control vs. treatment group).

In the curative protocol, both Diclofenac and CVE inhibited oedema over the course of the study (Figures 2(a) and 2(b)). Diclofenac decreased the total oedema of the control group which was 343.10 ± 6.71 by 37.25 ± 4.26%, 42.72 ± 7.16%, and 54.58 ± 4.45% at doses 10, 30, and 100 mgkg^−1^, respectively (Figure 2(c)). CVE also significantly inhibited total oedema compared to the control. Percentage inhibitions of total oedema by CVE were 22.72 ± 4.19%, 35.78 ± 4.51%, and 45.14 ± 4.60% at doses 30, 100, and 300 mgkg^−1^, respectively (Figure 2(d)).

### 4.3. Relative Potency of CVE

**Figure 3:**
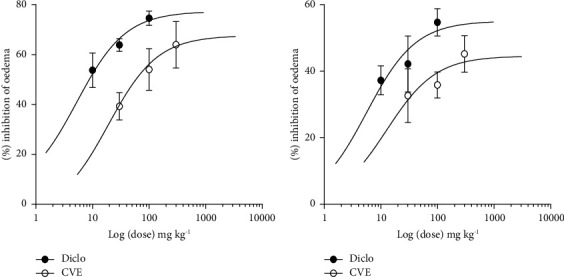
Dose-response curves for CVE (30–300 mgkg^−1^) and diclofenac (10–100 mgkg^−1^).

The potency of the extract was compared to that of diclofenac by estimating the ED_50_ values in each protocol (Figure 3). In the prophylactic study, the ED_50_ was 4.490 ± 1.48 and 22.78 ± 1.9 for diclofenac and CVE, respectively (Figure 3(a)). In the curative study, the ED_50_ was 5.60 ± 1.94 and 12.66 ± 2.51, respectively, for diclofenac and CVE (Figure 3(b)). In both protocols, diclofenac and CVE were effective in inhibiting oedema; however, diclofenac was found to be more potent than CVE.

### 4.4. Effect of CVE on Prostaglandin E_2_-Induced Oedema

**Figure 4:**
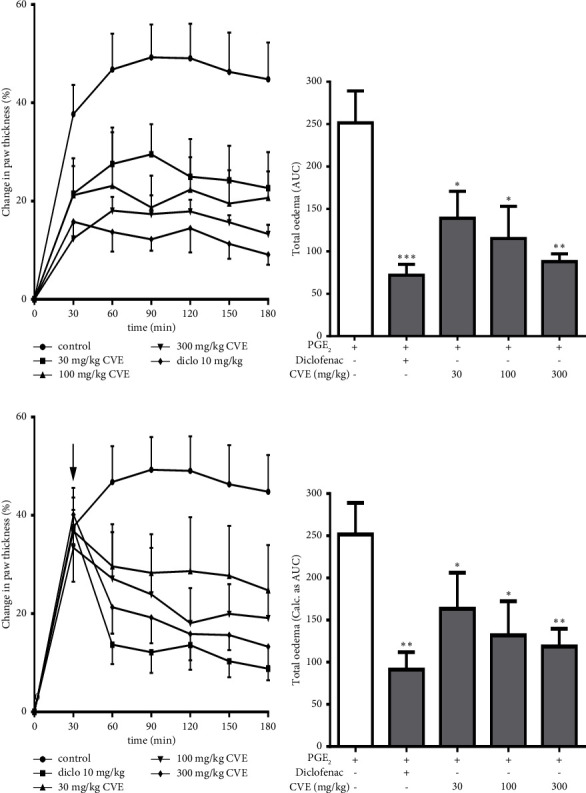
Effect of CVE on PGE_2_-induced paw oedema in ICR mice. (a, b) Mice (*n* = 5) were given saline (10 mlkg^−1^*p.o*), diclofenac (10 mgkg^−1^*p.o*), or CVE (30–300 mgkg^−1^*p.o*) 30 min before PGE_2_ injection. (c, d) Mice (*n* = 5) orally received saline (10 mlkg^−1^), diclofenac (10 mgkg^−1^), or CVE (30, 100, and 300 mgkg^−1^) 30 min after PGE_2_ injection. ^*∗*^*P* ≤ 0.05; ^*∗∗*^*P* ≤ 0.01; ^*∗∗∗*^*P* ≤ 0.001.

In the prophylactic study, the control mice exhibited the highest inflammatory response as measured over the 3 h period. The maximum change in paw thickness of the control mice was 49.26 ± 6.67%, and this occurred at the 90^th^ min, while diclofenac significantly reduced the paw thickness by 4-fold (12.21 ± 2.30%) (Figure 4(a)). Also, at this same time, CVE reduced the paw thickness by 2-fold (29.53 ± 6.12%, 18.71 ± 6.49%, and 17.37 ± 3.79%) at doses 30, 100, and 300 mgkg^−1^, respectively (Figure 4(a)).

Diclofenac inhibited total oedema by 71.09 ± 3.90%. CVE inhibited total oedema by 25.43 ± 32.36%, 51.85 ± 13.67%, and 55.42 ± 16.28% at doses 30, 100, and 300 mgkg^−1^, respectively (Figure 4(b)).

In the curative study, Diclofenac significantly reduced the AUC to 91.34 ± 20.59 (*P*=0.0059) (Figure 4(c)) and the percentage of inhibition was 61.29 ± 8.08%. CVE also reduced the total inflammatory response to 128.0 ± 37.24 (*P*=0.0477) and 118.8 ± 21.02 (*P*=0.0150), respectively, at doses 100 and 300 mgkg^−1^, albeit insignificant at 30 mgkg^−1^ (Figure 4(d)). Percentage inhibitions of total oedema by CVE were 7.99 ± 1.38%, 48.74 ± 12.11%, and 52.46 ± 2.64% at doses 30, 100, and 300 mgkg^−1^, respectively (Figure 4(d)).

### 4.5. Bradykinin-Induced Paw Oedema

**Figure 5:**
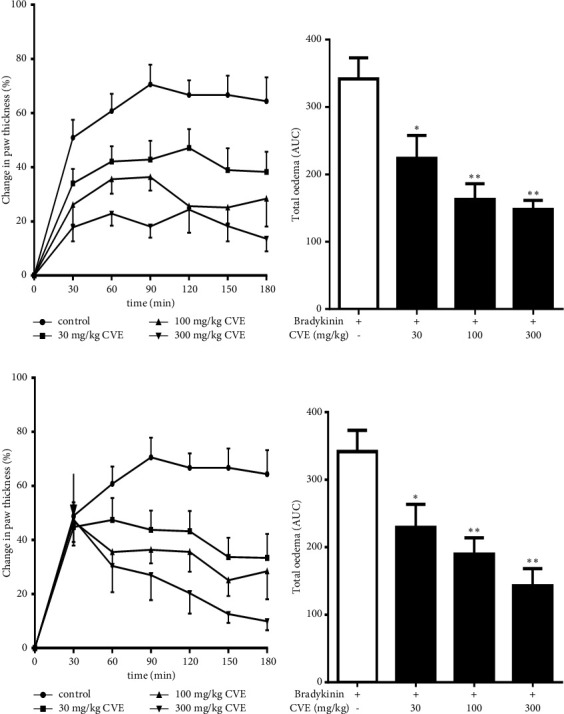
Effect of CVE on bradykinin-induced inflammation in ICR mice. Mice (*n* = 5) were injected with 1 *µ*g of 1 nM of the bradykinin subplantar thirty minutes after oral administration of saline (1 mlkg^−1^) or extract (30–300 mgkg^−1^). In the therapeutic protocol, CVE was given 30 min after bradykinin challenge. Results were presented as the mean ± SEM. Prophylaxis: (a) time-course effect (b) AUC. Curative: (c) time-course effect and (d) AUC. ^*∗*^*P* < 0.05; ^*∗∗*^*P* < 0.01.

Subplantar injection of 1 *µ*g bradykinin (10 nmol/paw) evoked acute inflammation characterized by oedema of the injected paw. The maximal oedema response of the control mice occurred on the 90^th^ min with increase in paw thickness to 70.57 ± 12.47% of the initial (Figure 5(a)). In contrast, pretreatment of mice with CVE significantly prevented inflammation induced by bradykinin. The percentage increases in paw thickness of the CVE-treated mice at the 90^th^ min were as low as 18.08 ± 3.82%, 36.37 ± 12.27, and 42.57 ± 17.92%, respectively, by 300, 100, and 30 mgkg^−1^ as against 70.57 ± 12.47% of the control. The total inflammatory response (AUC) of the control mice for 3 h was 341.86 ± 107.84. The percentage inhibitions of the total oedema of the control mice by the extract were 68.36 ± 23.62% (AUC = 108.15 ± 54.27), 52.22 ± 21.74% (AUC = 162.88 ± 62.18), and 34.53 ± 16.28% (224.12 ± 94.74), respectively, by 300, 100, and 30 mgkg^−1^ (Figure 5(b)).

In the curative protocol also, the extract dose dependently reduced bradykinin-induced inflammation in ICR mice. Treatment with CVE one hour after the bradykinin challenge significantly reduced the maximum inflammatory response of the control mice which occurred on the 90^th^ min. Changes in paw thickness in the CVE-treated mice at the 90^th^ min were 26.99 ± 9.38%, 36.37 ± 11.92%, and 43.76 ± 19.83% (*P* < 0.05) as against 70.57 ± 12.47% of the control (Figure 5(c)). The percentage inhibitions of the total inflammatory response (as AUC) by the extract were 58.52 ± 17.83%, 44.53 ± 19.72%, and 32.88 ± 17.38%, respectively, by 300, 100, and 30 mgkg^−1^ (Figure 5(d)).

## 5. Discussion

Carrageenan-induced oedema as described by Winter [18] is a widely used acute inflammatory model for screening novel compounds for anti-inflammatory activity. Carrageenan injection produced a rapidly developed biphasic acute inflammatory response characterized by swelling, heat, and pain [19]. In phase one, which occurs between 0 and 2 h, inflammatory mediators such as serotonin, nitric oxide, and histamine are released during the first 1 h, and then bradykinin release follows. Treatment of phase 1 with NSAIDs does not result in inhibition [20, 21]. The phase two which occurs between 2 and 5 h is mediated by the release of prostaglandin, and unlike phase 1, treatment of phase 2 with NSAIDs leads to significant improvement [22–24]. Diclofenac which was used as a reference drug in this study is known to inhibit cyclooxygenase 2 which releases prostaglandin from arachidonic acids in inflammatory cells [25, 26]. Similarly, CVE significantly prevented inflammation in both prophylactic and curative protocols. It could be possible that the anti-inflammatory activity of CVE is due to the inhibition of some of the above compounds especially prostaglandins which mediate inflammation.

Prostaglandin E_2_ (PGE_2_) is the most ubiquitous prostaglandin in the biological system. It is principally involved in inflammatory processes that lead to swelling, redness, heat, and pain [27]. Intradermal administration of PGE_2_ causes vasodilation which enhances accumulation of cells and effector molecules that result in oedema as observed in this study. Inflammatory response in phase one is due to direct activation of intradermal PGE_2_ receptors whereas the sustained response was due to subsequent synthesis of prostaglandins from tissue macrophages through the arachidonic pathway [27]. Unlike other proinflammatory mediators, this synthetic pathway of PGE_2_ is very sensitive to treatment with NSAIDs. Inhibition of PGE_2_ by NSAIDs, analgesic drugs, and steroid hormones produces marked clinical improvement in inflammatory, pyretic, and pain conditions. Bradykinin causes vasodilatation and increases vascular permeability through the B2 receptor. The inflammatory effect of bradykinin is due to its ability to elicit the release of vasodilators such as prostacyclin (PGI_2_) and nitric oxide (NO) [28]. Therefore, inhibition of the B2 receptor by a synthetic or organic compound could possibly prevent inflammation.

Anti-inflammatory effect of diclofenac in prophylactic and curative protocols is mainly by the inhibition of COX 2, as mentioned above. Inhibition of PGE_2_-induced oedema by CVE could possibly be due to direct blockade of PGE_2_ receptors or inhibition of further synthesis and/or release of PGE_2_ from macrophages [29, 30].

Anti-inflammatory effect of CVE in this study could be due to the various phytochemical ingredients found in the extract. For instance, flavonoids are widely known to exhibit anti-inflammatory, antioxidant, and free radical scavenging effects [31]. Alkaloids, terpenoids, tannins, and saponins are also known to possess health benefits including anti-inflammatory, anti-cancer, antiviral, antibiotic, antioxidants, and free radical scavenging effects [32, 33].

It could be inferred from this study that CVE has anti-inflammatory activity. However, further investigation is required to ascertain its mechanism of action.

## 6. Conclusion

We conclude that hydroethanolic extract of *Cordia vignei* leaf inhibits carrageenan-induced foot oedema in chicks and prostaglandin E_2_-induced paw oedema in mice.

## Figures and Tables

**Table 1:** Results of the phytochemical tests of CVE.

Phytochemical	Observation/color change	Inference
Saponin	Presence of froth	++
Tannin	Blue-black/green precipitate	++
Glycosides	Brown precipitate	+
Alkaloids	Orange–red spots/precipitate	++
Flavonoids	Yellowish coloration	++
Steroids	No colour change	−
Terpenoids	Reddish-brown	+

− not present; + light coloration/precipitation; ++ deep coloration/precipitation.

## Data Availability

The data supporting the conclusions of this article are available at Kwame Nkrumah University of Science and Technology Library repository and could be accessed through the corresponding author upon reasonable request.

## References

[1] Ash M. (2015). *Immune Dysfunction and Inflammation*.

[2] Rosas C. E., Correa L. B., Henriques M. G. (2017). *Neutrophils in Rheumatoid Arthritis*.

[3] Patil K. R., Mahajan U. B., Unger B. S. (2019). Animal models of inflammation for screening of anti-inflammatory drugs: implications for the discovery and development of phytopharmaceuticalsfiammation for screening of anti-infiammatory drugs: implications for the discovery and development of phytopharmaceuticals. *International Journal of Molecular Sciences*.

[4] Giles A. J., Hutchinson M. K. N. D., Sonnemann H. M. (2018). Dexamethasone-induced immunosuppression: mechanisms and implications for immunotherapy. *Journal for ImmunoTherapy of Cancer*.

[5] Henneh I. T., Akrofi R., Ameyaw E. O. (2018). Stem bark extract of sterculia setigera delile exhibits anti-inflammatory properties through membrane stabilization, inhibition of protein denaturation and prostaglandin E2 activity. *Journal of Pharmaceutical Research International*.

[6] Burkill H. M. (2004). *The Useful Plants of West Tropical Africa*.

[7] Owusu G., Obiri D. D., Ainooson G. K. (2020). Acetic acid-induced ulcerative colitis in sprague dawley rats is suppressed by hydroethanolic extract of Cordia vignei leaves through reduced serum levels of TNF-*α* and IL-6. *International Journal of Chronic Diseases*.

[8] Agyare C., Spiegler V., Asase A., Scholz M., Hempel G., Hensel A. (2017). An ethnopharmacological survey of medicinal plants traditionally used for cancer treatment in the Ashanti region, Ghana. *Journal of Ethnopharmacology*.

[9] Woode E., Amoh-Barimah A., Abotsi W. M., Ainooson G. A., Owusu G. (2012). Analgesic effect of stem bark extract of *Trichilia monadelpha* (Thonn) JJ De Wilde. *Indian Journal of Pharmacology*.

[10] Janet C. G., Barbee R. W., Bielitezki J. T. (2011). *Guide for the Care and Use of Laboratory Animals*.

[11] Sofowora A. (1993). *Phytochemical Screening of Medicinal Plants and Traditional Medicine in Africa*.

[12] Usman H., Abdulrahman F. I., Usman A. (2009). Qualitative phytochemical screening and in vitro antimicrobial effects of Methanol stem bark extract of ficus thonningii (moraceae). *African Journal of Traditional, Complementary and Alternative Medicines: AJTCAM*.

[13] Evans W. C. (2002). *Trease and Evans Pharmacognosy*.

[14] Houghton P. J., Raman A. (1998). *Laboratory Handbook for the Fractionation of Natural Extracts*.

[15] Adesegun S., Ayoola G., Coker H. (2008). Phytochemical screening and antioxidant activities of some selected medicinal plants used for malaria therapy in southwestern Nigeria. *Tropical Journal of Pharmaceutical Research*.

[16] Jana S., Shekhawat G. S. (2010). Phytochemical analysis and antibacterial screening of in vivo and in vitro extracts of Indian medicinal herb: anethum graveolens. *Research Journal of Medicinal Plant*.

[17] Fereidoni M., Ahmadiani A., Semnanian S., Javan M. (2000). An accurate and simple method for measurement of paw edema. *Journal of Pharmacological and Toxicological Methods*.

[18] Winter C. A., Risley E. A., Nuss G. W. (1962). Carrageenin-induced edema in hind paw of the rat as an assay for antiinflammatory drugs. *Experimental Biology and Medicine*.

[19] Lopes L. C., Ernesto de Carvalho J., Kakimore M. (2013). Pharmacological characterization of Solanum cernuum Vell.: 31-norcycloartanones with analgesic and anti-inflammatory properties. *Inflammopharmacology*.

[20] Mansouri M. T., Hemmati A. A., Naghizadeh B., Mard S. A., Rezaie A., Ghorbanzadeh B. (2015). A study of the mechanisms underlying the anti-inflammatory effect of ellagic acid in carrageenan-induced paw edema in rats. *Indian Journal of Pharmacology*.

[21] Korneev K. V., Atretkhany K. S. N., Drutskaya M. S., Grivennikov S. I., Kuprash D. V., Nedospasov S. A. (2017). TLR-signaling and pro-inflammatory cytokines as drivers of tumorigenesis. *Cytokine*.

[22] Nemeth Z. H., Bogdanovski D. A., Barratt-Stopper P., Paglinco S. R., Antonioli L., Rolandelli R. H. (2017). Crohn’s disease and ulcerative colitis show unique cytokine profiles. *Cureus*.

[23] Essel L. B., Obiri D. D., Osafo N., Antwi A. O., Duduyemi B. M. (2017). The ethanolic stem-bark extract of *Antrocaryon micraster* inhibits carrageenan-induced pleurisy and pedal oedema in murine models of inflammation. *International Scholarly Research Notices*.

[24] Necas J., Bartosikova L. (2013). Carrageenan: a review. *Veterinarni Medicina*.

[25] Thomazzi S. M., Silva C. B., Silveira D. C. (2010). Antinociceptive and anti-inflammatory activities of Bowdichia virgilioides (sucupira). *Journal of Ethnopharmacology*.

[26] Debprasad C., Hemanta M., Paromita B. (2012). Inhibition ofNO2,PGE2, TNF-*α*,and*i*NOS EXpression by*Shorea robusta*L.: an ethnomedicine used for anti-inflammatory and analgesic activity. *Evidence-based Complementary and Alternative Medicine*.

[27] Sun X., Li Q. (2018). Prostaglandin EP2 receptor: novel therapeutic target for human cancers (Review). *International Journal of Molecular Medicine*.

[28] Ricciotti E., FitzGerald G. A. (2011). Prostaglandins and inflammation. *Arteriosclerosis, Thrombosis, and Vascular Biology*.

[29] Pinsornsak P., Kanokkangsadal P., Itharat A. (2015). The clinical efficacy and safety of the Sahastara remedy versus diclofenac in the treatment of osteoarthritis of the knee: a double-blind, randomized, and controlled trial. *Evidence-based Complementary and Alternative Medicine*.

[30] Kim H. S., Kwon O. K., Park J. W. (2013). Anti-inflammatory activities of methanol extract of*Mastixia arborea*C.B. Clarke as to mouse macrophage and paw edema. *Bioscience, Biotechnology, and Biochemistry*.

[31] Kim W.-S., Choi W. J., Lee S. (2014). Anti-inflammatory, antioxidant and antimicrobial effects of artemisinin extracts from*Artemisia annua*L. *KOREAN JOURNAL OF PHYSIOLOGY and PHARMACOLOGY*.

[32] Debnath T., Kim D., Lim B. (2013). Natural products as a source of anti-inflammatory agents associated with inflammatory bowel disease. *Molecules*.

[33] Lee J. H., Choi E. J., Park H.-S., Kim G. H. (2014). Evaluation of Compositae sp. plants for antioxidant activity, antiinflammatory, anticancer and antiadipogenic activity*in vitro*. *Food and Agricultural Immunology*.

